# Visible expansion, fragile effective coverage: Using repeated eye health system assessments to interpret limited change in eye health outcomes in Sierra Leone, 2013–2025

**DOI:** 10.1016/j.dialog.2026.100347

**Published:** 2026-09-05

**Authors:** Stevens Bechange, Elena Schmidt, Iain Jones, Lloyd Harrison-Williams, Nazaradden Ibrahim, Tahir Bockarie, Tiangay Gondoe, Emma Jolley

**Affiliations:** a Sightsavers Sierra Leone Country Office, Freetown, Sierra Leone; b Sightsavers United Kingdom, Haywards Heath, UK; c Ministry of Health, Freetown, Sierra Leone; d Independent Consultant, Freetown, Sierra Leone

**Keywords:** Eye health systems, Avoidable blindness, Cataract surgical coverage, Documentary analysis, Health systems strengthening, Sierra Leone

## Abstract

**Purpose:**

To examine how reported changes in Sierra Leone's eye health system may help interpret limited change in avoidable blindness, cataract surgical coverage, and gender equity between repeated population eye health surveys.

**Methods:**

We reviewed four national reports: RAABs from 2010 and 2021 and EHSAs from 2013 and 2025. We extracted findings on governance, financing, workforce, service delivery, medical products, and information systems. We compared what changed and what persisted across the two EHSAs, assessed how comparable the reports were, and linked system themes to RAAB outcomes as plausible interpretations rather than direct causal effects.

**Results:**

Blindness among adults aged 50 years and older changed little, from about 4.9% in 2010 to 5.4% in 2021. Cataract surgical coverage rose from about 40.5% to 50.5%, but coverage fell slightly among women while rising among men. By 2025, eye health had stronger coordination, more district eye units, a larger workforce, wider equipment distribution, and eye indicators in DHIS2. These gains matched the modest rise in coverage. However, donor dependence, weak domestic financing, uneven workforce deployment, fragile procurement and maintenance, and weak routine data use remained.

**Conclusion:**

Repeated EHSAs can help explain why visible service expansion may not lead quickly to better population outcomes. In Sierra Leone, progress was real but too uneven and fragile to shift blindness more clearly or close the gender gap. Future gains will depend on stronger domestic financing, fairer workforce deployment, reliable supplies and maintenance, and routine equity monitoring.

## Highlights

• Repeated EHSA assessments can help explain slow improvements in population eye health outcomes.
• In Sierra Leone, cataract services expanded, but key system functions remained fragile.
• Gender gaps in cataract surgical coverage persisted, with limited sex-disaggregated data to explain them.
• Greater service visibility did not translate into effective or equitable coverage.

## 1 Introduction

Vision impairment and blindness remain major public health concerns, and much of this burden is avoidable through timely prevention, treatment, or rehabilitation [1], [2], [3]. Because major causes of vision loss, especially cataract and uncorrected refractive error, can often be addressed through known and cost-effective interventions, eye health outcomes are also useful indicators of how well a health system converts resources into effective care [4], [5], [6].

This policy relevance has been reinforced by the World Report on Vision [5], [7] and the agenda for integrated people-centred eye care within universal health coverage (UHC) [5]. From a health-systems perspective, the key issue is not only whether services exist, but whether financing, workforce, supplies, referral systems, and information systems work together well enough for people to obtain timely and affordable care. Eye health is therefore a useful tracer of system performance because it depends on coordination across several parts of the health system at the same time [2], [6].

Rapid Assessments of Avoidable Blindness (RAABs) are widely used to estimate the prevalence and causes of vision impairment and blindness among adults aged 50 years and older and to assess service coverage indicators such as cataract surgical coverage (CSC) [8], [9]. RAABs are valuable for policy and planning because they show whether services are reaching people in need [8]. However, they describe population outcomes; they do not, on their own, explain why progress is slow or why inequities persist.

Sierra Leone offers an important case for examining this question. The country has faced long-standing resource constraints, major health emergencies including Ebola and COVID-19, and continuing challenges in equitable service delivery [10], [11]. In such settings, services often expand through a mix of government action and external support, but this does not always produce strong or lasting system capacity [12], [13]. Sierra Leone also has a rare combination of repeated population eye health surveys and repeated eye health system assessments, creating an opportunity to examine outcome trends alongside reported system change over time.

RAABs from 2010 and 2021 suggest limited improvement in key eye health outcomes in Sierra Leone [14]. In this paper, we focus on three outcomes that are both policy relevant and plausibly linked to health system performance: blindness prevalence, cataract surgical coverage, and gender inequality in cataract surgical coverage. These indicators capture unmet need, service reach, and equity in access.

To interpret these outcome patterns, we draw on Eye Health System Assessments (EHSAs), structured reviews of how eye care services are organised and supported within a health system [15], [16]. EHSAs are commonly organised around the World Health Organisation (WHO) health-system building blocks: governance and stewardship, financing, workforce, service delivery, medical products and technologies, and health information systems [15], [16]. Although the building blocks approach does not capture every dynamic in a health system, it is useful here because it helps to show where constraints cluster, where improvements are partial, and how weaknesses in one part of the system can limit gains in another [17].

EHSAs are usually used as stand-alone planning tools. Much less attention has been given to what they can show when repeated and compared over time. Our contribution is to treat repeated EHSAs as longitudinal evidence on system change and to use them alongside repeated RAABs to explain why eye health outcomes improved only slowly. This allows us to examine path dependency in simple terms: once financing, workforce deployment, outreach patterns, and supply arrangements become established in a certain way, later reforms may improve parts of the system without fully changing how the system works overall [18].

We therefore use RAABs as outcome evidence and EHSAs as the main source for system analysis. The paper focuses on reported system strengths, weaknesses, and changes over time, and on how these help explain the observed RAAB patterns. Its specific novelty lies in showing how repeated EHSAs can be analysed as longitudinal system evidence, rather than used only as standalone planning documents, to interpret slow change in population eye health outcomes.

The study had two aims: 1) to examine how Sierra Leone's eye health system changed between the 2013 and 2025 EHSAs, and 2) to assess how these reported changes and persistent weaknesses help explain patterns in selected RAAB outcomes between 2010 and 2021. We asked: what strengths, weaknesses, and changes were reported across the health-system building blocks over time; and how do these findings help interpret limited change in blindness prevalence, modest change in CSC, and persistent gender inequality in CSC?

## 2 Methods

### 2.1 Study design

We conducted a longitudinal documentary analysis to compare repeated eye health system assessments over time and relate their findings to selected population eye health outcomes. The analysis combined RAAB reports from 2010 and 2021 with EHSA reports from 2013 and 2025. The aim was not to make causal claims about specific system changes and RAAB indicators, but to assess whether the strengths, weaknesses, and changes reported in the EHSAs help explain the observed RAAB patterns.

### 2.2 Conceptual framework

The analysis was organised using the WHO health system building blocks framework: governance and stewardship, financing, human resources for health, service delivery, medical products and technologies, and health information systems [19]. This framework was used because both the 2013 and 2025 EHSAs were structured around similar system domains and because it offers a practical way to compare how different parts of the system changed over time. We used it as an organising framework, while also paying attention to the way problems interacted across building blocks, how dependence on partners shaped implementation, and how repeated bottlenecks suggested underlying system fragility.

### 2.3 Data sources

We used four core documents (Table 1): the 2010 RAAB report [20], the 2021 RAAB report [14], the 2013 EHSA report [21], and the 2025 EHSA report, published in February 2026 [22]. The follow-up EHSA was conducted and analysed in 2025, although the final report was published in February 2026; we therefore refer to it as the 2025 EHSA when discussing the assessment period and cite the published report as a 2026 source. The RAAB reports were used to identify outcome patterns over time. The EHSA reports were the main analytic material for identifying reported system strengths, weaknesses, and changes. Where the EHSA reports referred to national policies, plans, or administrative arrangements, these references informed interpretation, but the main unit of analysis remained the EHSA report texts. The 2025 EHSA expanded upon the 2013 assessment by incorporating additional facility readiness mapping and focus group discussions with providers and service users, generating deeper insight into system functionality and bottlenecks.

**Table 1:** Key characteristics of the four core documents included in the analysis.

Document	Year	Role in the study	Type of information used
RAAB report	2010	Baseline outcome source	Blindness prevalence, cataract surgical coverage, and gender differences in cataract service access
EHSA report	2013	Baseline system assessment	Reported strengths, weaknesses, and bottlenecks across the six health-system building blocks
RAAB report	2021	Follow-up outcome source	Change over time in blindness prevalence, cataract surgical coverage, and gender inequality in coverage
EHSA report	2025	Follow-up system assessment	Reported reforms, persistent bottlenecks, implementation gaps, and selected empirical indicators across the six building blocks

### 2.4 Selection of outcomes and documents

We selected three RAAB outcomes for analysis: blindness prevalence, cataract surgical coverage, and gender inequality in cataract surgical coverage [23]. These outcomes were chosen because they were available across the two survey rounds and because they are plausibly influenced by the kinds of system factors described in the EHSAs, such as financing, workforce distribution, service organisation, outreach, referral systems, and affordability. We did not attempt to explain every RAAB indicator. Instead, we focused on a small set of outcomes that could be linked most clearly to reported system features.

The EHSA reports were included because they represent the two eye health system assessments available for Sierra Leone across the study period. Both reports assessed eye health services using a building blocks approach and drew on document review, facility assessments, and stakeholder interviews in purposefully selected geographical areas representing better and worse resources settings [21], [22]. We treated them as comparable for the main domains of system analysis, but not as identical studies. Before coding, we compared their aims, system domains, source types, geographical coverage, and level of detail. Where the 2025 EHSA contained more extensive facility readiness mapping or qualitative material than the 2013 report, we used those findings to deepen interpretation but did not treat their greater detail as proof of system change. Apparent changes were interpreted more cautiously when they could have reflected differences in reporting depth, emphasis, or documentation rather than a real change in services.

### 2.5 Document review, data extraction, and narrative synthesis

The EHSA reports were read in full and reviewed section by section. We extracted information on governance, financing, workforce, service delivery, medical products and technologies, and health information systems (Table 2).

**Table 2:** Data extraction framework.

Building block	Focus of extraction	Illustrative empirical details
Governance and leadership	Leadership structures, coordination, planning, supervision, accountability, and data use for governance	Policy and strategy status, district coordination arrangements, supervision structures
Financing	Funding sources, budget lines, donor roles, cost recovery, financial protection, fund flow, and accountability	Public health expenditure, out-of-pocket spending, transport costs, eye-health budget arrangements
Human resources for health	Cadres, numbers, training, deployment, retention, supervision, and volunteer roles	Numbers of ophthalmologists and ophthalmic nurses, proportion of volunteers, years without payroll status
Service delivery	Service availability, outreach, referral, surgery, specialised care, integration, and continuity of care	Spread of eye units, cataract surgical capacity/coverage, district service capacity
Medical products and technologies	Equipment availability, maintenance, medicines, consumables, supply chain, and assistive technologies	Distribution of basic ophthalmic equipment, stock-outs, donor procurement, repair delays
Health information systems	Reporting tools, DHIS2 integration, data quality, feedback loops, M&E capacity, and data use	Eye indicators in DHIS2, use of paper forms, local data review practices

Within each building block, we coded reported strengths, bottlenecks, reforms, and implementation gaps, then compared the 2013 and 2025 assessments to identify continuity, change, and persistent weakness (Table 3).

**Table 3:** Narrative synthesis matrix comparing the 2013 and 2025 EHSAs.

Building block	2013	2025	Interpretive summary
Governance and leadership	Weak policy base, limited formal coordination, strong partner influence	Formal policy and strategy in place, stronger NEHP visibility, more structured district coordination	Governance became more formalised, but system authority remained limited
Financing	Heavy donor dependence, minimal domestic support, weak fund flow	Continued donor dependence, small government contribution, weak district financial autonomy	Financial sustainability changed little
Human resources for health	Extreme shortages, no in-country ophthalmology training, urban concentration	Modest workforce growth, local training started, continued maldistribution and high volunteer dependence	Capacity increased, but deployment and retention remained weak
Service delivery	Limited geographic coverage, ad hoc outreach, weak referral and follow-up	Wider service presence, more regular surgery in some government hospitals, outreach still donor-reliant	Access improved, but continuity and equity remained fragile
Medical products and technologies	Very limited equipment outside a few sites, donor-led supply, no maintenance system	Better equipment distribution, but uneven supply, frequent stock-outs, weak repair capacity	Procurement improved more than long-term service readiness
Health information systems	Paper-based, fragmented, weak standardisation, little use for planning	Core eye indicators integrated into DHIS2, but continued reliance on paper tools and weak feedback	Digital foundations improved, but routine data use remained weak

We also extracted selected empirical findings where these helped anchor the narrative, such as workforce numbers, district coverage, funding patterns, and service delivery indicators. Using a narrative synthesis approach [24], we organised findings first within each building block, then across building blocks, and finally mapped them to the selected RAAB outcome patterns (Table 4) to assess whether reported system changes were likely to be sufficient, too limited, too uneven, or too fragile to produce stronger improvement over time.

**Table 4:** Analytic linking framework used to interpret RAAB outcomes.

RAAB outcome	Relevant building blocks	Interpretive question	Expected type of explanation
Blindness prevalence	Service delivery; human resources; financing; medical products and technologies	Were services available at sufficient scale, continuity, and quality to reduce avoidable blindness over time?	Why burden changed little despite some service expansion
Cataract surgical coverage	Service delivery; workforce; financing; governance	Did surgery become more available, affordable, and organised across districts and referral pathways?	Why coverage improved only modestly
Gender inequality in cataract surgical coverage	Financing; service delivery; governance; health information systems	Did the system reduce access barriers for women and monitor equity in service reach?	Why service expansion did not produce equal gains for women and men

### 2.6 Data analysis

Analysis proceeded in three linked stages. First, key characteristics of the four core documents were extracted into a summary table to show their year, role in the study, and type of information used (Table 1). Second, the two EHSA reports were imported into Nvivo 15 [25] and coded using a thematic analysis approach [26]. Coding was guided by the study question: how do reported strengths, weaknesses, and changes in the eye health system help interpret limited change in blindness prevalence, modest change in cataract surgical coverage, and persistent gender inequality in cataract surgical coverage? Initial coding was descriptive and stayed close to the report text, focusing on issues such as staffing gaps, donor dependence, weak supervision, district coordination, stock-outs, affordability and service continuity. Related codes were then grouped into broader interpretive categories within and across the six health-system building blocks, including partial formalisation, persistent centralisation, fragile service expansion, weak system readiness, limited use of data for decision-making and weak equity monitoring. Third, we compared coded findings between the 2013 and 2025 EHSAs and then linked these themes to the selected RAAB outcome patterns. We treated this linkage as an interpretive association, not as proof that a specific system feature caused a specific RAAB result. We also separated 2025 EHSA findings that appeared to describe conditions likely to have existed before or during 2021, such as long-standing workforce maldistribution, donor dependence, and weak procurement, from findings likely to represent more recent developments, such as newer policy, training, or reporting arrangements. The analysis did not estimate effect sizes or test the independent effect of each building block. NVivo 15 supported transparent organisation of the coded material, while the final synthesis focused on whether recurring system patterns were broad enough, sustained enough, and equitable enough to plausibly explain the RAAB trends.

For gender and sex reporting, we followed the principle of the SAGER guidelines [27] by checking whether the source documents reported sex-disaggregated information on service use, workforce composition, affordability, and barriers to access. The RAAB reports provided sex-disaggregated cataract surgical coverage, which was one of the main outcomes for this article. The EHSAs contained some discussion of access barriers but did not consistently report sex-disaggregated system data. We therefore interpreted gender inequality cautiously and noted this gap as part of the evidence base rather than treating it as a fully measured system pathway.

### 2.7 Ethics considerations

Ethical approval was not required for this study because it was based on secondary analysis of existing reports and did not involve direct contact with human participants. However, all four original studies used in this analysis, the two RAABs and the two EHSAs, had ethical clearance from the national ethics board in Sierra Leone.

## 3 Results

We first summarise the selected RAAB outcome patterns, then compare the 2013 and 2025 EHSAs across the six health system building blocks. Findings are presented with a specific interpretive purpose: to show how reported system changes may help make sense of limited change in blindness prevalence, modest improvement in CSC, and persistent gender inequality in CSC. The building blocks are not treated as separate problems. Financing shaped workforce deployment, outreach, supplies, maintenance, and supervision; weak data use limited the system's ability to target gaps; and uneven service delivery meant that expansion did not benefit all groups equally. Because the most recent RAAB was conducted in 2021 and the follow-up EHSA was completed in 2025 and published in February 2026, we distinguish, where possible, between long-standing system features that probably shaped the 2010 to 2021 outcome trends and newer developments that may only affect future outcomes.

### 3.1 Selected RAAB outcome patterns

The RAABs show limited change in the main outcome indicators between 2010 and 2021 [14]. Blindness prevalence among adults aged 50 years and older remained broadly stable, at about 4.9% in 2010 and 5.4% in 2021. Cataract surgical coverage increased only modestly, from about 40.5% to 50.5%. Gender inequality also persisted: CSC decreased from about 34.3% to 33.2% among women, compared with an increase from about 49.3% to 68.3% among men, showing that women benefited less than men from gains in cataract service access (Table 5). These outcome patterns provide the comparative benchmark for the system findings below.

**Table 5:** Selected quantitative changes in outcomes and system readiness.

Indicator	Earlier time point	Later time point	What this shows for the storyline
Blindness prevalence among adults aged 50 years and older	4.9% in 2010	5.4% in 2021	Little population-level improvement despite service expansion
Overall cataract surgical coverage	40.5% in 2010	50.5% in 2021	Modest improvement in access to cataract surgery
Cataract surgical coverage among women	34.3% in 2010	33.2% in 2021	No clear improvement for women
Cataract surgical coverage among men	49.3% in 2010	68.3% in 2021	Men benefited more from service gains
Total eye health personnel	66 in 2013	97 in 2025	Workforce grew, but shortages and maldistribution remained
Ophthalmologists	3 in 2013	6 in 2025	Numbers doubled but remained far below need
Ophthalmic nurses	41 in 2013	68 in 2025	Mid-level capacity increased
District hospitals with eye units	Fewer than 10 in 2013	All district hospitals by 2025	Geographic presence improved
Eye indicators in DHIS2	Not integrated in 2013	Core indicators integrated by 2025	Reporting improved, but data use remained weak

Table 5 allows comparison of outcome and system indicators side by side. It also makes the main interpretation more transparent: service capacity increased, but the gains were not reliable, sustained, or equitable enough to produce larger improvements in population outcomes.

### 3.2 Governance and leadership

Governance was stronger and more formalised in 2025 than in 2013, but it remained overly centralised and only partly able to steer the wider system. At the national level, the National Eye Health Programme (NEHP) had gained credibility, sustained institutional memory despite leadership changes, and moved eye health away from ad hoc donor projects towards a nationally endorsed policy and strategy. Partner coordination had also become more formal through periodic reviews, Ministry planning processes, and an eye health technical working group. At district level, leadership had evolved from informal and reactive arrangements in 2013 to more regular coordination between DHMTs and hospital eye units in districts such as Bo, Tonkolili, Moyamba, and Makeni, where outreach plans, patient data, and logistics were now discussed more routinely. These changes help to explain why cataract services became more organised and why CSC improved at least modestly between the RAAB rounds. However, the main governance constraints also persisted. Decision making over budgets, training, and staff deployment remained centralised, and district eye focal persons often lacked formal influence within DHMT structures, and there was still no strong mechanism to help the NEHP influence cross-cutting areas such as human resources, pharmaceuticals, and financing. Partner engagement had broadened since 2013, but government ownership remained partial because donors still shaped many operational decisions. In relation to the RAAB findings, this suggests that governance reforms were sufficient to improve coordination and service visibility, but not strong enough to drive the deeper and more even system change needed to reduce blindness prevalence more clearly or to adress gender inequality systematically across districts.

### 3.3 Financing

The financing picture changed very little between 2013 and 2025, despite some administrative development. In both assessments, eye care depended heavily on external support to finance training, outreach, equipment, consumables, and supervision. By 2025, the donor base had narrowed, with Sightsavers remaining the main predictable funder. Government support continued to cover mainly salaries, utilities, and health facility maintenance, while the dedicated budget line for eye health remained too small to finance routine medicines, consumables, equipment, or outreach. At health facility level, cost recovery helped to keep services running, but these funds were inconsistent, weakened by poverty, and limited by the large share of patients receiving free or subsidized care through the Free Health Care Initiative. Several financing weaknesses identified in 2013 therefore remained in place: funds were still centralised through the NEHP, district autonomy remained low, disbursement delays were common, and financial reporting specific to eye care was still weak. The broader fiscal context also remained restrictive: public expenditure on health was reported at 8.5% of GDP in 2013 and 4.7% in 2023, while out-of-pocket spending accounted for 55% of total health expenditure and social health protection coverage remained below 1% of the population. In relation to the RAAB findings, this financing pattern helps to explain all three outcome trends. It helps to explain why blindness prevalence changed little, because service continuity, medicines, outreach, and follow-up remained financially fragile; why CSC improved only modestly, because surgery and outreach could expand but not at the scale needed; and why gender inequality likely persisted, because direct and indirect costs continued to fall heavily on households, especially older and poorer women.

### 3.4 Human resources for eye health

By 2025, the eye health workforce was larger, more diverse, and better linked to national training efforts than it had been in 2013, but it remained the weakest building block of the system. Total eye health personnel increased from 66 in 2013 to 97 in 2025. This included growth in ophthalmologists from 3 to 6, ophthalmic nurses from 41 to 68, optometrists from 0 to 5, optometrist technicians from 3 to 6, and the addition of 12 ophthalmic community health officers. In-country ophthalmology training had also started through residency at Connaught Hospital, and since 2019 the WHO AFRO Primary Eye Care training toolkit had been rolled out with about 20 master trainers to strengthen capacity at the PHC level. These gains help explain why more districts were able to run eye units, outreach, and cataract services than in 2013. However, workforce growth did not keep pace with population need. Using the IAPB benchmark of 4 ophthalmologists (or surgeons) per million population in Africa, Sierra Leone had a gap of about 21 ophthalmologists in 2013 and about 28 in 2025 because the population had grown to about 8.5 million while only 6 ophthalmologists were available. Distribution also remained highly unequal. Most trained staff were concentrated in Freetown, Bo, and Kenema, while remote districts continued to rely on visiting teams, outreach, or very small eye units. Volunteers remained central to service delivery, making up about 40% to 80% of the workforce in visited facilities, with some serving for more than seven years without payroll status. Training, supervision, and career progression also remained fragmented and largely donor supported, while retention was weakened by migration, poor incentives, irregular salaries, and limited recognition. These patterns suggest that workforce growth was enough to support some increase in CSC, but too small, too unevenly distributed, and too unstable to generate larger reductions in blindness prevalence or more equitable gains in access.

### 3.5 Service delivery

Service delivery was more widespread and somewhat better organised in 2025 than it had been in 2013, but it remained uneven, donor reliant, and weak in continuity of care. Eye units were reported in all district hospitals by 2025, compared with fewer than ten a decade earlier, and the number of facilities offering cataract surgery had increased beyond the seven reported in the earlier assessment. Government hospitals such as Bo, Kenema, and Makeni were now performing regular cataract surgeries, marking a shift away from the earlier concentration of surgery in faith-based hospitals. Outreach had also become more structured and more closely linked to district planning, with DHMTs playing some coordinating role in districts such as Bo, Kenema, and Port Loko. These changes are consistent with the RAAB finding of modest improvement in cataract surgical coverage. However, many of the main service delivery constraints identified in 2013 remained. Northern and remote districts were still underserved, outreach continued to depend on donor funding for transport, consumables, and field allowances, and many rural populations still relied on intermittent campaigns as their main point of access. The hub-and-spoke referral model had improved coordination between PHUs and secondary hospitals, but it remained only partly institutionalised, and referral and feedback systems were still weak. Cataract service output had improved but remained far below need: the country reported only 409 cataract surgeries per million population in 2015, far below the Vision 2020 benchmark of 2000 per million for Africa. Specialised services also expanded only slightly. Glaucoma diagnosis was available in most hospitals, but surgery remained limited to a few centres, while refraction and low-vision services had grown through 12 vision centres but remained sparse and concentrated in urban areas. In relation to the RAAB outcome patterns, this suggests that service delivery improved enough to expand reach, but not enough in scale, continuity, referral, follow-up, or rural access to produce larger reductions in blindness prevalence or to close gender and geographic gaps in cataract service use.

### 3.6 Medical products and equipment

Medical products and technologies were more widely available in 2025 than in 2013, but the supporting systems for equipment maintenance, supply, and equitable distribution remained weak. Most secondary hospitals now had basic ophthalmic equipment such as slit lamps, ophthalmoscopes, retinoscopes, sterilizers, and B-scan machines, whereas in 2013 only a few hospitals had functioning ophthalmic devices and even major hospitals lacked key items. By 2025, procurement decisions were no longer entirely ad hoc, and some eye equipment had entered national procurement planning. However, important weaknesses remained. PHUs and smaller district hospitals often lacked essential tools, some remote facilities worked with only one functioning diagnostic device, and equipment distribution did not always match patient load or staff skills. Equipment maintenance was a major unresolved bottleneck. Few biomedical technicians were trained in ophthalmic equipment, repairs often depended on visiting technicians from Freetown or donor support, and broken machines could remain out of service for months. Medicines and consumables showed a similar pattern. Compared with 2013, there was modest progress, including retention of a few ophthalmic medicines on the national Essential Medicines List, but most eye drops, ointments, sutures, dyes, and anti-glaucoma medicines were still supplied through donor channels and stock-outs remained common. Evidence from primary care also suggested a major readiness gap: in a 2021 to 2022 survey of 32 PHC facilities in four districts, none had the full set of eye care supplies and consumables recommended in the WHO Primary Eye Care manual, and medicine availability was especially poor in Bonthe. Access to spectacles and low vision aids also remained limited, with most facilities lacking on-site refraction, glazing, or fitting, and spectacles often costing 50 to 70 New Leones. In relation to the RAAB findings, these supply and maintenance weaknesses help explain why broader service availability did not translate into stronger outcome gains. Facilities may have existed, but unreliable equipment, medicine shortages, weak primary care readiness, and limited visual aids reduced the effective quality and continuity of care needed to lower blindness prevalence more clearly or expand cataract and refractive services equitably.

### 3.7 Health information systems

Eye health information systems were better integrated into national reporting structures in 2025 than they had been in 2013, but they remained weak in data quality, feedback, and practical use. The main improvement was the inclusion of core eye indicators in DHIS2, which gave eye care greater national visibility than in 2013, when reporting was fragmented, paper-based, and often reduced to broad categories such as eye infection. By 2025, more facilities were routinely recording eye health specific data, monthly reporting was occurring in districts such as Bo, Makeni, and Pujehun, and some units were using local records to review patient trends, outreach uptake, and surgical activity. However, the system still depended heavily on manual registers and paper forms. Health Form 13, the eye-specific reporting tool, was often unavailable, forcing facilities to use general forms that grouped multiple eye conditions into a single category and reduced detail. Data validation remained inconsistent, discrepancies between facility registers and DHIS2 persisted, and feedback to facilities was still irregular. As in 2013, most data were collected for reporting rather than used actively for planning, budgeting, procurement, or workforce decisions. Monitoring and evaluation (M&E) and data review processes also remained heavily partner supported, while digital capacity was constrained by limited computers, internet access, and dedicated eye care M&E staff. Research and links with national performance structures had improved since 2013, and a future DHIS2 upgrade was expected to strengthen ophthalmic reporting, but routine data governance remained weak. In relation to the RAAB outcomes, this means that the system became better at seeing activity than at using data to change performance. Better reporting may have supported some growth in service organisation, but weak use of data for targeting, supervision, quality improvement, and equity monitoring likely limited the system's ability to accelerate cataract surgical coverage, reduce persistent blindness burden, or respond to gender differences in access.

## 4 Discussion

This study used repeated eye health system assessments to interpret limited change in eye health outcomes in Sierra Leone over time. The main finding is the gap between visible service expansion and fragile effective coverage. The system improved in structure, organisation, and geographic presence, and these gains are consistent with the modest improvement in cataract surgical coverage between the two RAAB rounds. Governance became more formalised, eye units spread across districts, the workforce grew, equipment became more available, and eye indicators entered DHIS2. At the same time, the core conditions needed to turn these gains into stronger outcomes remained weak. Financing stayed donor dependent, workforce deployment remained uneven, supply and maintenance systems remained fragile, and data were not routinely used to manage quality or equity. This combination of partial progress and persistent bottlenecks offers a plausible interpretation of why blindness prevalence changed little and why gender inequality in cataract surgical coverage persisted.

This finding is consistent with wider eye health literature showing that service availability alone is not enough to achieve effective coverage [6], [9], [15], [28]. Work on effective cataract surgical coverage and effective refractive error coverage has emphasised that access, quality, affordability, and equity need to be considered together [9], [29], [30]. In Sierra Leone, the increase in eye units, staff, equipment, and reporting structures suggests that service availability improved. However, the system conditions needed to convert this availability into effective coverage remained weak. This helps make sense of why cataract surgical coverage improved only modestly and why blindness prevalence did not fall more clearly.

The findings also reflect wider evidence from health systems research in low-resource settings [31], [32]. Programmes can expand activity and make services more visible while leaving deeper system functions underdeveloped [33]. Donor-dependent financing, centralised decision-making, fragile supply chains, weak maintenance systems, and uneven workforce deployment can all limit whether reforms become routine practice [34], [35]. Sierra Leone's eye health system illustrates this problem clearly. Expansion occurred, but much of the system still depended on external support, local improvisation, and weak feedback systems.

A key message from the longitudinal comparison is that service expansion alone was not enough. The 2025 EHSA describes a system with more eye units, more regular district coordination, some in-country specialist training, broader equipment distribution, and stronger reporting structures than in 2013. These improvements are consistent with the modest rise in cataract surgical coverage. At the same time, the timing of change matters. Some of the positive changes reported in the 2025 EHSA may have been introduced only recently, including after 2021 or too close to the 2021 RAAB for their effects to be visible in population-level outcomes. This means that some reforms may already be improving service provision even if their impact was not yet measurable in the 2021 survey. However, the same reforms remained too partial, uneven, and fragile to shift outcomes more substantially. Outreach still depended on donor support for transport, consumables, and allowances. Government financing remained too limited to sustain routine operations, while public expenditure on health had fallen to 7.95% of GDP in 2022 and out-of-pocket spending remained high at 55% of total health expenditure. Equipment was more available, but many facilities still faced stock-outs, equipment repair delays, or dependence on a single functioning diagnostic device. In practical terms, the system became better at making services visible, but not reliable enough to deliver care at the scale, continuity, and equity needed for a clearer reduction in blindness burden [12].

The findings also show that weaknesses across building blocks combined to limit effective coverage. More trained staff did not fully solve access problems because they were still too few and too concentrated in urban areas. Wider service availability did not guarantee treatment because medicines, consumables, and functioning equipment were often unreliable, especially outside larger hospitals. Better reporting did not automatically improve performance because data were still used weakly for targeting, supervision, and equity monitoring. Financing constraints affected all these areas at once, limiting outreach, maintenance, supervision, and continuity of care. This pattern helps to explain the overall RAAB findings: a modest increase in cataract surgical coverage without a corresponding system wide shift large enough to reduce blindness prevalence more clearly [9].

The gender findings add an important equity dimension. Previous evidence shows that women often face greater barriers to cataract services, including costs, distance, transport, household responsibilities, and limited control over resources [36], [37], [38]. In this study, the RAABs showed that women did not benefit equally from improvements in cataract surgical coverage. The EHSAs suggest plausible system pathways, including weak affordability, reliance on intermittent outreach, poor follow-up, and limited equity monitoring. However, the EHSAs did not consistently report sex-disaggregated system data on service utilisation, workforce composition, affordability, referral completion, or surgical uptake outside the RAAB findings. The study therefore cannot identify the precise mechanisms behind the gender gap. This absence of sex-disaggregated system evidence is itself an important finding, because it limits the system's ability to monitor whether reforms are reaching women and men equally.

The study also adds to methodological debates about how to understand slow or unequal progress in population health outcomes when routine data are incomplete [39], [40], [41]. RAABs provide valuable outcome evidence, but they do not explain why services succeed or fail. EHSAs are usually used for planning, but our analysis shows that, when repeated, they can help identify which system constraints persist, which reforms are recent, and which improvements may be too fragile to affect population outcomes. This approach may be useful in other settings where repeated population surveys exist, but routine service data are weak.

We were cautious in interpreting the link between the EHSA and RAAB findings. The 2025 EHSA cannot be treated as a direct explanation for outcomes measured in the 2021 RAAB, because some reforms may have occurred after the survey or too close to it to affect population outcomes. We therefore gave more weight to long-standing system features, such as donor dependence, workforce maldistribution, weak procurement, poor equipment maintenance, and limited data use, when interpreting the 2010 to 2021 outcome trends. More recent changes, including newer policy, training, and reporting arrangements, are better understood as possible foundations for future improvement.

This study has a few limitations. First, it is a documentary and interpretive analysis based mainly on what was reported in the EHSA documents, rather than on a prospectively designed longitudinal dataset. Second, although the 2013 and 2025 assessments were similar enough in scope and purpose to support structured comparison, they may have differed in depth, emphasis, methods, and reporting style. To reduce this risk, we compared the reports by common system domains, gave greater weight to findings that appeared in both assessments or were supported by specific empirical detail, and avoided interpreting extra detail in the 2025 report as change unless there was clear evidence that the system itself had changed. Third, the timing of change is important and cannot be assessed precisely with the available documents. Some of the positive changes described in the 2025 EHSA may have been introduced only recently, including after 2021 or too close to the 2021 RAAB to affect the survey findings. Long-standing features such as donor dependence, workforce maldistribution, weak procurement, poor equipment maintenance, and limited routine data use are more relevant for interpreting the 2010 to 2021 RAAB trends, while newer policy, training, and reporting arrangements may be more relevant for future assessments. Fourth, the EHSAs did not consistently report sex-disaggregated system data on service utilisation, workforce composition, affordability, or referral and follow-up, which limited our ability to examine the system pathways behind gender inequality in cataract surgical coverage. Fifth, the building blocks framework was useful for organising the analysis, but it simplifies relationships within the system and does not fully capture politics, informal practice, or wider social determinants [42], [43]. Finally, the paper identifies plausible explanations for the RAAB patterns, but it does not test causal effects or quantify the independent contribution of each building block. The findings should therefore be read as a reasoned interpretation of repeated system evidence, not as proof of direct causation.

### 4.1 Conclusions and policy implications

The repeated EHSAs and RAABs suggest that Sierra Leone's eye health system progressed, but not enough in the functions most likely to shift population outcomes. The system became more organised and more visible, and these gains are likely to have supported modest improvement in CSC. However, financing, workforce distribution, supply chain reliability, equipment maintenance, follow-up, and equity monitoring remained too weak to convert service expansion into clearer reduction in blindness prevalence or into more equitable access to cataract surgery. The wider lesson is that health system strengthening should not be judged only by whether services, staff, equipment, or reporting tools have increased. These changes matter, but they may not improve population outcomes unless they are reliable, financed, equitably distributed, and used to solve practical access barriers [44]. The main lesson is that population eye health outcomes depend not only on whether services exist, but on whether the wider system can support them consistently, affordably, and fairly.

Several policy implications follow. Further expansion of service activity alone is unlikely to be enough. Greater attention is needed to the system conditions that turn service presence into effective coverage. This includes stronger domestic financing for essential eye-care services, consumables, maintenance, and spectacles; better workforce deployment, supervision, and retention; stronger integration of eye care into procurement, maintenance, and routine information systems; and monitoring that goes beyond activity counts to track coverage, quality, and equity. Without these changes, future gains may remain visible in service activity but limited in population outcomes [45].

This study also has methodological implications. Repeated system assessments can be used not only for planning, but also for understanding why outcomes do (or do not) change over time. In settings where routine data are incomplete, comparing repeated assessments offers a practical way to identify which weaknesses persist and which reported improvements may still be too small, too uneven, or too fragile to shift population eye health outcomes. Future research should test which changes are most likely to close the gap between service expansion and effective coverage. Priorities include using routine data to monitor gender and district equity, assessing the costs and effects of transport or outreach support for women and rural populations, examining models for retaining and deploying eye health workers outside larger towns, and evaluating whether procurement and maintenance reforms improve service continuity and surgical output.

## Declaration of generative AI and AI-assisted technologies in the manuscript preparation process

During the preparation of this work, we utilized NVivo's inbuilt AI assistant to support querying of data after coding, through matrix and other repetitive search patterns. The AI features were applied as an aid to enhance efficiency, while all interpretations remained researcher driven.

## CRediT authorship contribution statement

**Stevens Bechange:** Writing – review & editing, Writing – original draft, Validation, Software, Project administration, Methodology, Investigation, Formal analysis, Data curation, Conceptualization. **Elena Schmidt:** Writing – review & editing, Methodology, Investigation, Formal analysis, Data curation, Conceptualization. **Iain Jones:** Writing – review & editing, Methodology, Formal analysis, Data curation, Conceptualization. **Lloyd Harrison-Williams:** Writing – review & editing, Methodology, Formal analysis, Data curation, Conceptualization. **Nazaradden Ibrahim:** Writing – review & editing, Methodology, Formal analysis, Data curation, Conceptualization. **Tahir Bockarie:** Writing – review & editing, Methodology, Investigation, Formal analysis, Data curation, Conceptualization. **Tiangay Gondoe:** Writing – review & editing, Investigation, Formal analysis, Data curation, Conceptualization. **Emma Jolley:** Writing – review & editing, Methodology, Formal analysis, Conceptualization.

## Disclaimer

The findings and conclusions in this article are those of the authors and do not necessarily represent the official views of Sightsavers or the Sierra Leone Ministry of Health.

## Funding

This study was supported by funding from 10.13039/100009099Irish Aid.

## Declaration of competing interest

The authors declare that they have no known competing financial interests or personal relationships that could have appeared to influence the work reported in this paper.

## Data Availability

This study used secondary data from existing RAAB and EHSA reports. The reports cited in the reference list are available through government, institutional, or partner websites where public links are provided. No new primary data were generated for this analysis.
